# Supplementary material for: Splice-Junction-Based Mapping of Alternative Isoforms in the Human Proteome
Source: Cell Rep. Author manuscript; Available in PMC 2020 Jan 15. (PMC6961840; doi:10.1016/j.celrep.2019.11.026)
Supplement: 3 [file NIHMS1546469-supplement-3.zip › DF2/PXD009737/TestisTrypsin-210-Q5GLZ8-GMLADIPVTICTYPFVFDAQAK.pdf]

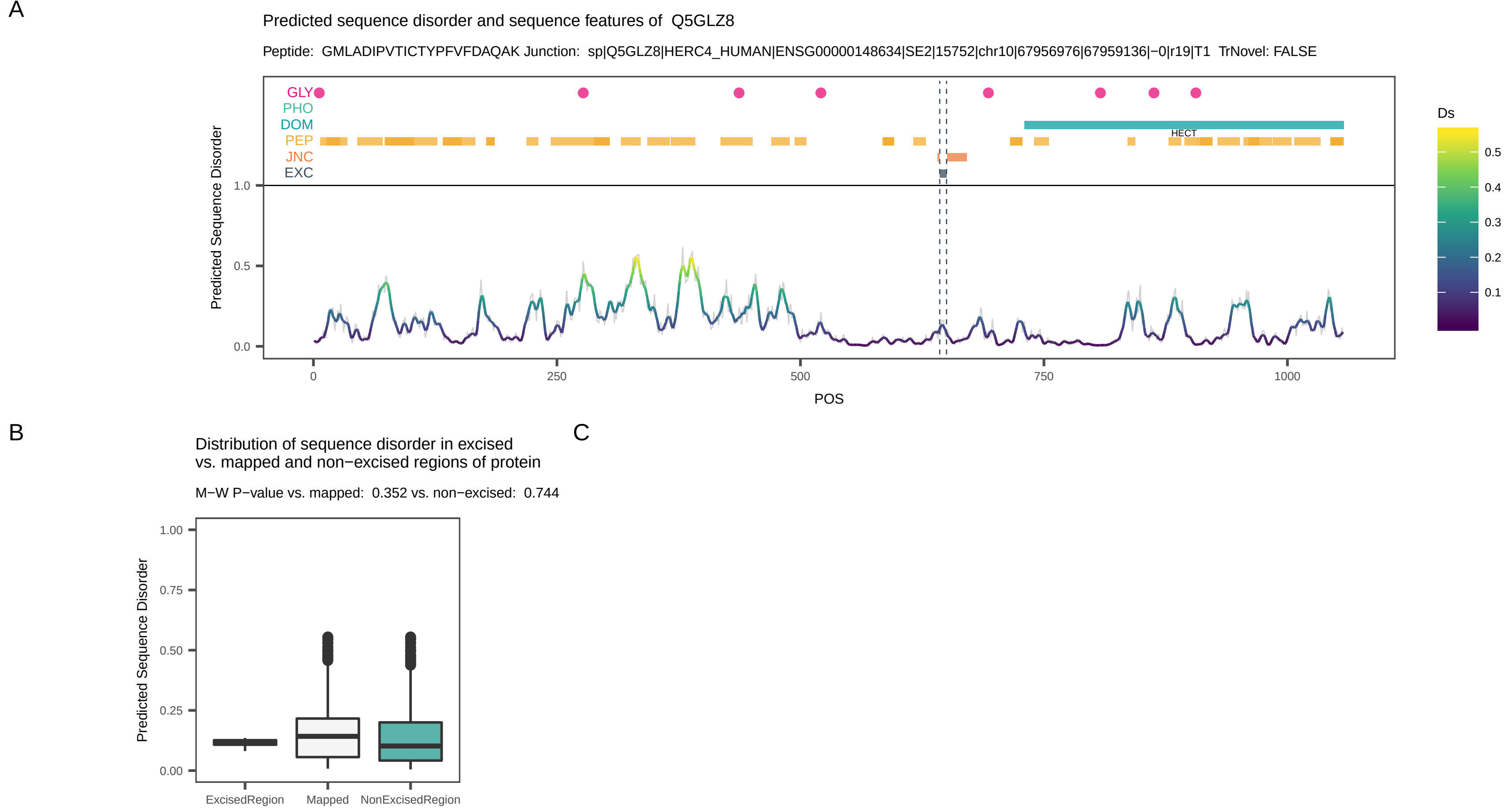
A
Predicted sequence disorder and sequence features of Q5GLZ8
Peptide: GMLADIPVTICTYPFVFDAQAK Junction: sp|Q5GLZ8|HERC4_HUMAN|ENSG00000148634|SE2|15752|chr10|67956976|67959136|−0|r19|T1 TrNovel: FALSE
GLY
PHO
DOM
PEP
JNC
EXC
HECT
Predicted Sequence Disorder
1.0
0.5
0.0
0
250
500
750
1000
POS
Ds
0.5
0.4
0.3
0.2
0.1
B
Distribution of sequence disorder in excised vs. mapped and non−excised regions of protein
M−W P−value vs. mapped: 0.352 vs. non−excised: 0.744
Predicted Sequence Disorder
1.00
0.75
0.50
0.25
0.00
ExcisedRegion
Mapped
NonExcisedRegion
C
